# SVM+KF Target Tracking Strategy Using the Signal Strength in Wireless Sensor Networks

**DOI:** 10.3390/s20143832

**Published:** 2020-07-09

**Authors:** Xing Wang, Xuejun Liu, Ziran Wang, Ruichao Li, Yiguang Wu

**Affiliations:** 1 Key Laboratory of Virtual Geographic Environment (Nanjing Normal University), Ministry of Education, Nanjing 210023, China; 181301021@stu.njnu.edu.cn (X.W.); wangzr@nnutc.edu.cn (Z.W.); 191335015@stu.njnu.edu.cn (R.L.); 131301021@stu.njnu.edu.cn (Y.W.); 2 State Key Laboratory Cultivation Base of Geographical Environment Evolution (Jiangsu Province), Nanjing 210023, China; 3 Jiangsu Center for Collaborative Innovation in Geographical Information Resource Development and Application, Nanjing 210023, China

**Keywords:** wireless sensor network, target tracking, support vector machine, Kalman filtering

## Abstract

Target Tracking (TT) is a fundamental application of wireless sensor networks. TT based on received signal strength indication (RSSI) is by far the cheapest and simplest approach, but suffers from a low stability and precision owing to multiple paths, occlusions, and decalibration effects. To address this problem, we propose an innovative TT algorithm, known as the SVM+KF method, which combines the support vector machine (SVM) and an improved Kalman filter (KF). We first use the SVM to obtain an initial estimate of the target’s position based on the RSSI. This enhances the ability of our algorithm to process nonlinear data. We then apply an improved KF to modify this estimated position. Our improved KF adds the threshold value of the innovation update in the traditional KF. This value changes dynamically according to the target speed and network parameters to ensure the stability of the results. Simulations and real experiments in different scenarios demonstrate that our algorithm provides a superior tracking accuracy and stability compared to similar algorithms.

## 1. Introduction

Target tracking (TT) refers to the development and utilization of mobility models to determine information about the movement (such as the position, speed, and direction) of a target. This constitutes a fundamental and challenging application of wireless sensor networks (WSNs) [1,2,3]. One of the criteria with which TT algorithms in WSNs can be classified is whether the targets are cooperative with the tracking system. These are referred to as active and passive modes [4,5], respectively, and in this paper, we focus on the problem of active TT. Based on the premise that the locations of the nodes in the network are known a priori [4,5,6,7], existing TT algorithms attempt to achieve localization and estimate the trajectories of targets by interpreting several metrics of the transmissions from the target’s tag, such as received signal strength indication (RSSI) [8], angle of arrival [9], time difference of arrival [10], and time-of-arrival [11]. Among them, the RSSI-based techniques have been extensively investigated since (a) they are available in most of the commercial wireless devices and (b) they do not need any additional sensors or hardware customization [8,12]. RSSI-based TT usually includes three main stages: target detection, target location, and target trajectory prediction [13,14,15]. In active TT, the latter two processes are of primary importance [16,17,18,19].

The target localization process typically utilizes the radio propagation path-loss model to infer the distance of the target from a node. Usually, this model is applied by assuming that either the channel is a perfect free-space medium, or that extensive channel measurements and modeling are performed in the deployment of the system. However, dynamic outcomes in the environment, such as non-line-of-sight (NLOS), signal attenuation, and multi-path propagation, can affect signal propagation and consequently compound the challenges for target tracking [15]. Consequently, RSSI-based TT is either not feasible in many practical applications, or can only be achieved through an extensive set of experimental channel measurements [20]. While some researchers have been working on more robust path-loss models that consider these factors in their features, these models are usually very complex and only applicable to specific environments [13,20].

In the trajectory prediction process, Bayesian framework-based filters are the dominant approach [21]. Classical filters include the Kalman filter (KF) [14], extended KF (EKF) [22], unscented KF (UKF) [23], and particle filter (PF) [24]. Among them, KF provides an optimal statistical solution in scenarios with linear models and white noise. However, the accuracy and stability are difficult to guarantee in practical applications using KF-based methods. Furthermore, many problems in the real world cannot be represented by linear models. While the EKF, UKF, and other derivatives of KF attempt to address these problems, their adaptability for tracking in the real world still requires further improvement [25,26]. On the other hand, while PF can flexibly adapt to the nonlinear dynamic model and multimodal observation model, the particle degradation phenomenon leads to a decline of the tracking accuracy [25,27]. 

The above limitations exhibited by existing methods indicate that precise calculation of the nonlinear mapping relationship between the RSSI and the target position is key for stable and accurate TT results. Nevertheless, despite these shortcomings, KF—due to its smaller computational load and low-storage requirements—is still a popular and efficient recursive method commonly used for RSSI-based TT [28]. However, the trajectory prediction reliability of KF in nonlinear scenarios constitutes the “bottleneck” in its development in real-world applications, and therefore requires further development. 

Recent work has adopted a learning-by-example (LBE) approach [12,24,29,30] to address the aforementioned problems, in which the relationship between the RSSI value and the distance is a more sophisticated function. LBE systems are usually composed of two phases, in which one incorporates offline training and the other online testing. During the training phase, the features of the signal received at the network nodes are stored concurrently with the known position of the target to build a database of input–output relationships. After training has been conducted using the above database, pattern matching algorithms (e.g., support vector machine (SVM), neural network (NN), and k-nearest neighbor) are then applied in the testing phase to establish the unknown locations of the targets and plot the target trajectory combined with the filtering methods. LBE systems suitably capture the sophisticated relationship between the RSSI behavior and the target position, while avoiding complex path-loss model formulas [31]. Furthermore, SVM, a classic pattern matching method, has been found to exhibit advantageous features for TT in terms of its ability to solve nonlinear and high-dimensional pattern recognition problems with a small number of samples, avoiding “dimensionality disasters” and “over study” problems [29]. 

In this study, we build on this recent work to propose an innovative LBE algorithm, known as the SVM+KF algorithm, which improves the stability and accuracy of tracking results. The new algorithm combines the SVM and KF methods, and consists of offline model training and online target tracking phases. In the offline model training phase, the database, preprocessed by density-based spatial clustering of application with noise (DBSCAN), is used with the SVM algorithm to define a kernel-based model, whose input is the RSSI value and output is the corresponding position. In the online tracking phase, a first position estimate is obtained with the use of the already defined SVM model and the measured RSSI values. This estimate is then corrected by an improved KF based on innovation sequence modification to achieve a better tracking performance. 

The rest of the study is organized as follows. In Section 2, we provide a review of related LBE TT algorithms. The details of our proposed methodology are described in Section 3. Various simulation and experimental results are presented in Section 4 to study the performance of the proposed algorithm. Finally, the conclusions are summarized in Section 5.

## 2. Related Work

Numerous research studies attempting to deal with the dynamic nature of RSSI-based TT have been reported in the WSN literature [30,31,32,33]. In this section, we restrict our review to studies that are relevant to LBE-based methods, on which our approach is based. LBE methods combine pattern matching algorithms and filtering methods, and we cover these two aspects in our review. 

While numerous filtering algorithms have been extensively used in LBE-based TT for WSNs, KF has particularly attracted much attention as a classic filtering algorithm. However, KF is prone to instability. One approach that can be employed to avoid the instability of traditional KF and offer superior tracking performances, proposed by Wang et al. [32], is to improve the noise model by incorporating both additive noises and multiplicative noises in distance sensing. Furthermore, they use the maximum likelihood estimator for prelocalization of the target and measurement conversion to remove the measurement nonlinearity. These converted measurements and their associated noise statistics are then used in a standard KF for a recursive update of the target state. Although this method is effective and easy to implement, the cost of the system setup and maintenance is high. Chi et al. [34] used an extreme learning machine (ELM) to improve the estimation accuracy and the robustness of traditional KF for tracking in WSNs. Mahfouz et al. [20] trained the ridge regression (RR) and the vector-output regularized least-squares algorithms off-line to obtain a first estimation position. In the on-line tracking process, after the RSSIs and instantaneous acceleration of a moving target are measured, a first position estimate is obtained by using the already-defined kernel-based model. This estimate is combined with the acceleration information, by means of a KF, to achieve a better accuracy. Simulation results show that their algorithm can achieve robust tracking results when the acceleration information or the RSSI measures are affected by noise. However, multiple filters and multiple sensors are used to detect and TT, resulting in a high energy consumption. Taking the speed information of the target as a factor is an effective method for improving the accuracy of the tracking result, which is also employed in our method. Similar to these methods, other LBE methods using KF as the filter have also been developed, with differences in the various pattern matching (prelocalization) methods that are employed. 

In addition to KF, other relevant filtering algorithms have also been adopted. Ahmadi et al. [35] used the regression tree algorithm (RT) to estimate the target position using the RSSI, and Bayesian filtering, such as traditional KF, and PF to enhance their results. They found that this combination provided a good tradeoff between accuracy and robustness. However, the implementation of this algorithm in real-time WSN is complex. Jondhale et al. [36] used the generalized regression neural network (GRNN) to obtain initial location estimates of the target’s motion, which were then modified by KF and UKF respectively to improve the tracking performance. This method has also been applied for TT using Bluetooth and smartphones [37]. The above implementations have not yet been tested and verified in terms of their performance for variation in the measurement noise. In addition, Shi et al. [33] proposed a new localization strategy that combined hidden Markov models and EKF to identify sight conditions and mitigate NLOS errors. In their algorithm, HMM parameters are obtained by off-line training, with sight conditions as the hidden state and quantized RSSI measurements as observations. The sight conditions are then identified by an on-line forward-only algorithm. However, this method may be unsuitable for some cases where the target has a clear moving direction rather than random motion. Besides, this method lacks generalization in NLOS scenarios caused by dynamic factors. By combining the estimated sight conditions, the target is located by EKF to achieve the real-time localization. Wang et al. [38] proposed a polynomial fitting-based adjusted KF (PF-AKF) method in a WSN framework to alleviate the NLOS effect. PF-AKF employs polynomial fitting to accomplish both NLOS identification and distance prediction. It then processes the measurements with adjusted KF (AKF), conducting weighted filters in the case of the NLOS condition. Simulation and experimental results obtained from a real indoor environment demonstrate the superior performance of their method. 

In terms of pattern matching algorithms, many have been involved in the above examples, such as RR, GRNN, and ELM. For our method, we utilize SVM, which is a machine learning method based on statistical learning theory that has been extensively used in Bluetooth locations, WiFi locations, and WSNs, because of its numerous advantages in solving issues related to small samples, nonlinearity, and high-dimensional classification and prediction. Other LBE methods have also employed SVM for pattern matching.

Zhao et al. [29] used SVM to compute a classification boundary for TT, and then used KF to update this classification boundary in each sampling period. In this approach, the sampling points were classified by the updated classification line to calculate the coordinates of the corresponding observation points, which were then used to estimate the positions of the different targets. Simulation results validated the effectiveness and stability of their algorithm in comparison with existing methods. However, this combination of SVM and KF focused more on target classification during tracking. Furthermore, Liu et al. [39] proposed a distributed PF (DPF) based on the combination of SVM and DPF. However, they employed SVM to estimate the density in order to compress the particles to find the global optimum, and yield a sparse solution, rather than to estimate target coordinates. However, the performance of this method cannot approach the performance of centralized PF. Lam et al. [40] proposed a novel solution to improve RSSI-based distance estimation for smart object interaction applications in the IoT ecosystem. Their algorithm implements a KF on the edge to deal with noisy RSSI measurements and an optimized SVM on the cloud for distance estimation. Practical experiments verified that their algorithm can improve the performance in terms of the delay and accuracy. Nevertheless, in real-world situations, the targets (smartphones or users) are moving, and the number of packets received is limited. Therefore, the accuracy of this method is mainly affected by the signal receiving rate because a precise distance needs to be computed with a few samples.

While the aforementioned research provides important references and guidance for our work, and some scholars have attempted to address the problems of RSSI-based target positioning, ranging, and tracking by coupling SVM and KF [29,39,40], an LBE method based on combining SVM and KF has not yet been proposed. In contrast with existing methods, we propose the SVM + KF algorithm to improve the stability and accuracy of tracking results and validate the effectiveness and stability of the algorithm via experiments and simulations. Our research aims to provide a useful supplement to RSSI-based TT in WSNs.

## 3. Target Tracking Algorithm

**Figure 1:**
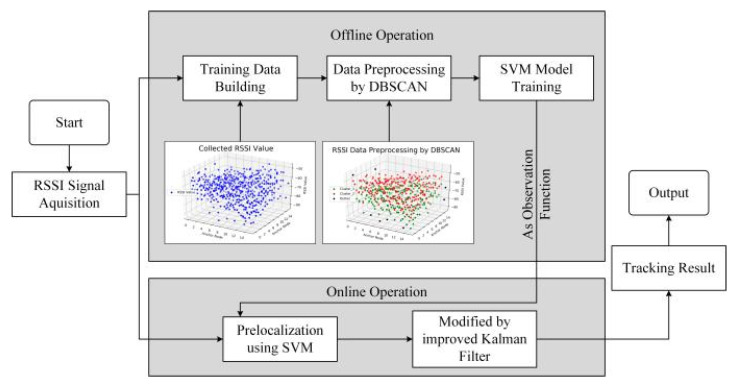
Structure of our algorithm.

In this section, we discuss our proposed SVM + KF algorithm in detail. SVM + KF combines SVM’s ability of nonlinear expression and KF’s advantages of low computation and memory requirements to achieve a better tracking performance. The implementation of SVM + KF consists of two phases: offline model training (discussed in Section 3.1) and online target tracking (discussed in Section 3.2). The structure of our algorithm is shown in Figure 1.

### 3.1. Offline SVM Model Training

As outlined in the previous section, we chose to employ SVM in our methodology, as SVM has been found to be suitable in numerous location-related applications (i.e., WiFi and Bluetooth) due to its advantages in handling small samples, nonlinearity, and high-dimensional classification and prediction problems. In this section, we introduce the training details of the SVM model during offline operations.

#### 3.1.1. Preprocessing of Training Data

In the SVM + KF method, the object positions at each time instant and the signals received by the nodes quantified by the RSSI are saved as training data. We trained the SVM model once offline to exploit the relationship between the unknown object positions and the RSSI values for the subsequent successive and online prediction (testing phase). No other a priori information on the scenario is required to perform the data fitting process (training phase).

Assuming that $A=\left\{a_{1},a_{2},\cdots ,a_{n}\right\}$ is the set of reference nodes that can be scanned in the entire localization area, the RSSI acquired by any subset $A_{i}$($A_{i}\subseteq A$) can be expressed as $\left[(x_{i},y_{i}),r_{i}\right]$, where $\left(x_{i},y_{i}\right)$ are the two-dimensional (2D) coordinates of $A_{i}$ in the sampling place, $r_{i}=(RSSI_{i1},RSSI_{i2},\cdots ,RSSI_{im})$, and $RSSI_{ij}$ is the RSSI value of the jth (j≤m≤n) reference node in set $A_{i}$. 

Since the quality of the training data can considerably affect the SVM model training, we first preprocessed the training data, in order to eliminate the outliers in the RSSI data. Outlier detection in WSNs can be categorized into five main classes, namely, statistical, nearest neighbor, clustering, classification, and spectral decomposition [41,42]. We chose to use DBSCAN [43], a density-based, unsupervised, simple, and efficient clustering algorithm, proposed by Martin Este et al. in 1996 [44], to improve the quality of the training dataset based on the following three reasons:

- Burst noise can cause RSSI outliers at points/segments that are not adjacent to any other region. DBSCAN can discover and remove these outliers, while concurrently allowing their clustering without forcibly assigning them to any class;
- DBSCAN determines the number of classes by the tightness of the data distribution without the need to specify them in advance. This is crucial for our work because we cannot determine which signals contain burst noise, and we cannot know the number of classes in advance;
- DBSCAN can discover clusters of arbitrary shapes, and does not increase the number of classes because of an unconventional training data distribution, thus affecting subsequent judgments. Since it is hard to guarantee a regular distribution with our training data, clustering them with DBSCAN can significantly reduce the location error generated by the data distribution.

#### 3.1.2. Prelocalization Using SVM 

After $\left[(x_{i},y_{i}),r_{i}\right]$ is preprocessed by DBSCAN, it is used as a training dataset for the SVM model. Typically, classification partitioning of the dataset containing RSSI values is nonlinear as a function of the region. SVM maps the dataset to a higher-dimensional space through nonlinear mapping and identifies a partitioning hyperplane in this higher dimensional space. Suppose that the equation of the hyperplane used to classify different categories of samples in the higher dimensional space is

(1) $$wr^{T}+b=0,$$

where w and b are vectors. 

According to the structural risk minimization principle, the calculation of the optimal classification hyperplane can be transformed to solve a constrained maximum problem, as expressed by Formula (2).

(2) $$\{\begin{array}{l} \min(\frac{1}{2}{\|w\|}^{2})\\ s.t.y_{i}(wr_{i}^{T}+b)\geq 1\begin{array}{cc} & i=1,2,\cdots ,n \end{array} \end{array}$$

With soft-margin decision optimization and inner operator kernel functions, Formula (2) can be transformed into the following optimization problems:

(3) $$\{\begin{array}{l} \min(\frac{1}{2}{\|w\|}^{2}+C\frac{1}{2}\sum_{i=1}^{n}\xi_{i}^{})\\ \begin{array}{l} s.t.y_{i}[w^{T}\;•\;\varphi (x_{i})+b]\;\geq \;1-\xi_{i}^{},i=1,2,\cdots ,l\\ \begin{array}{l} \xi_{i}^{}\;\geq \;0,i=1,2,\cdots ,l\\ C>0 \end{array} \end{array} \end{array},$$

where w is the weight coefficient, C is the penalty factor, $\xi_{i}^{}$ is the slack variable, $\varphi (x_{i})$ is the nonlinear mapping from the input space to a high-dimensional space, and b is the optimal hyperplane offset.

By solving the dual problem of Formula (3), a nonlinear decision function can be obtained, as shown in Formula (4):

(4) $$f(x)=\mathrm{sgn}\left[\sum_{i=1}^{n}y_{i}a_{i}K(x_{i},x_{j})+b\right],$$

where, $a_{i}$ is the Lagrange multiplier and $K(x_{i},x_{j})$ is the corresponding inner product kernel function that satisfies Formula (5).

(5) $$K(x_{i},x_{j})=\phi {\left(x_{i}\right)}^{T}\phi (x_{j})$$

Different kernel functions form different nonlinear classification models for the same input space. The mainstream kernel function is as shown in Formula (6). Considering that the RSSI values collected in the real application are often nonlinear and non-Gaussian, the Radial Basis Function (RBF) is chosen as the kernel function.

(6) $$K(x_{i},x_{j})=\exp(-\gamma {\left\|x_{i}-y_{i}\right\|}^{2}),\gamma >0$$

**Figure 2:**
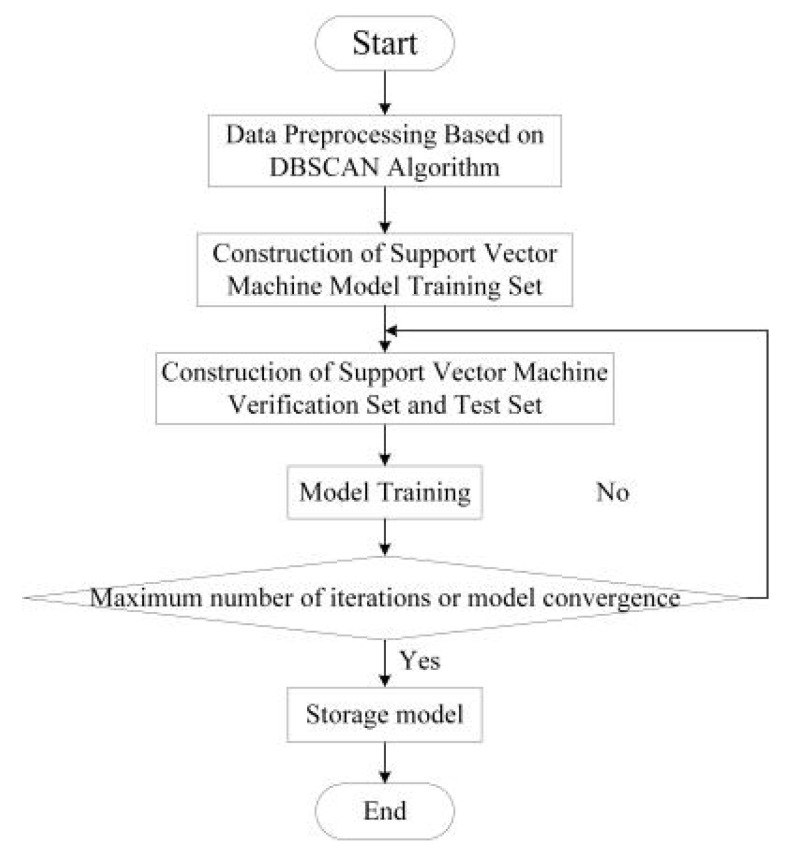
Flowchart for offline support vector machine (SVM) model training.

By solving Formula (6), the target location results can be obtained by the RSSI-based SVM. The optimal combination (C,γ) is determined by model training. Subsequently, the optimal SVM model is saved and used for subsequent operations of the method. The flowchart of the offline SVM model training is illustrated in Figure 2.

### 3.2. Online Target Tracking

For the online tracking process, the pre-located results obtained by the trained SVM model are used as the input to the KF to describe the target motion trajectory. Considering the shortcomings of traditional KF in real scenarios, we propose a new KF algorithm to improve the accuracy and stability of the tracking results based on innovation modification.

#### 3.2.1. KF Model Building

KF is a popular and efficient recursive method used to fuse low-level redundant data, i.e., it first state predicts the next state based on prior information and updates the prediction based on current observations. Using a set of inaccurate measurements observed over time, KF produces estimates that are more accurate than isolated measurements. The KF receives statistically optimal estimates for systems that can be described by a linear model, and the error can be represented as white noise [13,27].

To simplify the expression, we name the SVM positioning model equation X(k) the observation equation to build the KF. According to the basic theory of KF [45], a state–space model consisting of a state equation X(k) and an observation equation Y(k) is used to describe a dynamic system based on the following equations (for the target motion model, details are listed in Section 4.1):

(7) X(k+1)=ΦX(k)+W(k),

(8) Y(k)=HX(k)+V(k),

where

- k is discrete time, and $X(k)\in R^{n}$ and $Y(k)\in R^{m}$ are the state and RSSI observation equations at time k, respectively;
- $W(k)\in R^{n}\sim Ν(0,R^{n})$ is the random vector noise whose probability distribution is assumed to be the normal distribution with a zero mean and covariance matrix Q;
- $V(k)\in R^{m}\sim Ν(0,R^{m})$ is the observation noise with a normal distribution, zero mean, and covariance matrix R, and Φ is the state transition matrix that relates the current position of the target to its previous one;
- H is the observation matrix that relates the state X(k) to the measurement Y(k), and n and m are the dimensions of the matrix.

If Q and R are white noise signals and uncorrelated, the relationship between the above parameters can be expressed by Formula (9):

(9) $$\{\begin{array}{l} E(W(k))=0\\ E(V(k))=0\\ E(W(k)W^{T}(j))=Q\delta_{kj}\\ E(V(k)V^{T}(j))=R\delta_{kj}\\ E(W(k)V^{T}(j))=0 \end{array},$$

where E is a function used to identify the mean, k and j are arbitrary numbers, $\delta_{kk}=1$, and $\delta_{kj}=0$.

Suppose W(k) and V(k) are uncorrelated to the initial state X(0), which is

(10) $$E(X(0))=\mu_{0},$$

(11) $$E[(X(0)-\mu_{0}){\left(X(0)-\mu_{0}\right)}^{T}]=P_{0}.$$

The essence of KF is to relate the state variables at a certain moment to the measured value at the current time, and to solve the estimation of the linear minimum variance $\overset{\wedge }{X}(j|k)$ of the current state X(j) in some optimal way. The performance evaluation index of $\overset{\wedge }{X}(j|k)$ is as follows:

(12) $$J=E\left[{\left(X(j)-\overset{\wedge }{X}(j|k)\right)}^{T}(X(j)-\overset{\wedge }{X}(j|k))\right].$$

The KF’s deduction process is as follows,

(13) $$\tilde{X}(k)=\Phi \overset{\wedge }{X}(k-1)+W(k),$$

(14) $$\tilde{P}(k)=\Phi \overset{\wedge }{P}(k-1)\Phi^{T}+Q,$$

(15) $$\varepsilon (k)=Y(k)-H\tilde{X}(k),$$

(16) $$K(k)=\tilde{P}(k)H^{T}{\left(H\tilde{P}(k)H^{T}+R\right)}^{-1},$$

(17) $$\overset{\wedge }{X}(k)=\tilde{X}(k)+K(k)\varepsilon (k),$$

(18) $$\overset{\wedge }{P}(k)=(I_{n}-K(k)H)\tilde{P}(k),$$

where

- $\tilde{X}(k)$ and $\overset{\wedge }{X}(k)$ are the predicted and estimated values of the state variables at time k, respectively;
- $\tilde{P}(k)$ and $\overset{\wedge }{P}(k)$ are the covariance matrices of the prediction and the estimation errors of the state variable at time k, respectively;
- ε(k) is the innovation value corresponding to the observation matrix Y(k), $I_{n}$ is a unit matrix of order n, and K(k) is the Kalman gain at time k. In theory, $\varepsilon (k)\sim N(0,H_{k}\tilde{P}(k)H_{k}^{T}+R)$ is normally distributed white noise.

#### 3.2.2. Improved KF Based on Innovation Modification

The efficiency of target tracking mainly depends on the target state transition matrix. If the target transition matrix (Φ) closely resembles the movement of the target, then efficiency can be achieved. In the case of a maneuvering target, it becomes very difficult to track and predict the next location of a target. KF default uses the content velocity model and hence fails in the case of maneuvering target tracking. Additionally, KF gives a poor performance in the case of non-Gaussian Noise.

Since the traditional KF method has certain application limitations, we propose a new KF algorithm to improve the precision and stability of the trajectory. We know that the innovation in Formula (15) contains the model information and observation value of the system that can be used as an indicator to determine whether the estimated state value and the observed value are consistent [46]. In the fully-closed KF, the error caused by the system model can be controlled so that it is within a small range by feedback correction. Therefore, its influence can be ignored. Accordingly, the abnormality of the observed value (coordinate estimated by SVM) can be effectively reflected by the change of the statistical value of the innovation. 

Based on the above analysis, in the KF recursion process, we set the innovation ε(k) so that it is greater than the threshold $E_{Threshold}$ to be overwritten by the previous innovation value ε(k−1), and the current iterative operation is then performed. Therefore, Formula (17) is changed to (19).

(19) $$\overset{\wedge }{X}(k)=\{\begin{array}{cc} \tilde{X}(k)+K(k)\varepsilon (k) & \varepsilon (k)\leq E_{Threshold}\\ \tilde{X}(k)+K(k)\varepsilon (k-1) & \varepsilon (k)>E_{Threshold} \end{array}$$

(20) $$\begin{array}{cc} \varepsilon (k)=\varepsilon (k-1) & \left(\varepsilon (k)>E_{Threshold}\right) \end{array}$$

$E_{Threshold}$ is related to many factors and changes dynamically. $E_{Threshold}$ needs to be set according to the interference factors in the real world (e.g., the maneuverability characteristics of the target and NLOS). If $E_{Threshold}$ is too large, it can only reduce the effect of the interference factors on the positioning result to a small extent, resulting in a low filtering efficiency. If $E_{Threshold}$ is too small, the effective location is incorrectly discarded. This affects the accuracy of the tracking results. 

In this study, the determination of $E_{Threshold}$ considers the speed of the target’s motion, the sampling frequency, and the communication range of nodes. The specific deduction process is as follows.

**Figure 3:**
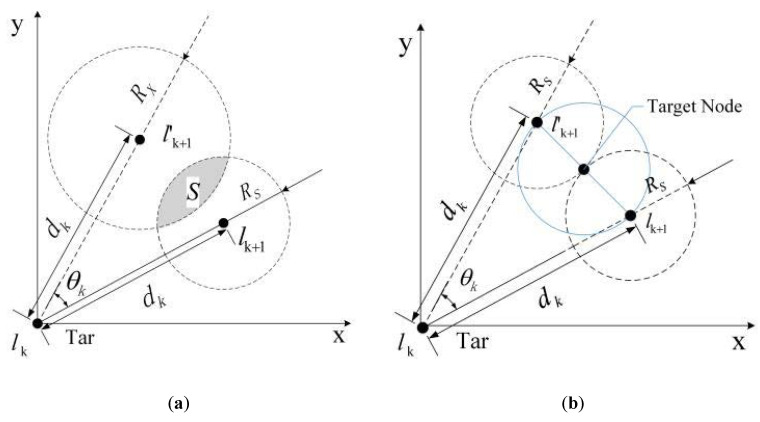
Target maneuver model. (**a**) Target maneuver model in general; (**b**) Target maneuver model under extreme conditions.

Assume that N nodes with the same communication radius $R_{S}$ are randomly distributed in a square region with a fixed area of $L_{1}\times L_{2}$. Figure 3 illustrates the target maneuver model. As shown in Figure 3a, $l_{k}(x_{k},y_{k})$ is the true position of the moving target tar A at time $t_{k}$. If a target maneuver occurs at time $t_{k}$, then $l_{k+1}^{}(x_{k+1}^{},y_{k+1}^{})$ and $l_{k+1}^{\prime }(x_{k+1}^{\prime },y_{k+1}^{\prime })$ are the true and predicted positions of tar A at time $t_{k+1}$, respectively. The deflection angle of $l_{k+1}^{}$ relative to $l_{k+1}^{\prime }$ is $\theta_{k}$. When the sampling interval $\Delta t_{k}\to 0^{+},(\Delta t_{k}=t_{k+1}-t_{k})$, the target speed change can be ignored, that is, $l_{k}^{}l_{k+1}^{}\approx l_{k}^{}l_{k+1}^{\prime }$. The rate of tar A at time $t_{k}$ is defined as $v_{k}$ and the sampling frequency of node as $f_{k}$. Accordingly, the displacement within time interval $\Delta t_{k}$ is

(21) dk=vkfk=lklk+1≈lklk+1′.

The distance between the true position for tar A and the predicted position at time $t_{k+1}$ is

(22) lk+1lk+1′=2dksin(θk2)=2vksin(θk2)fk.

**Figure 4:**
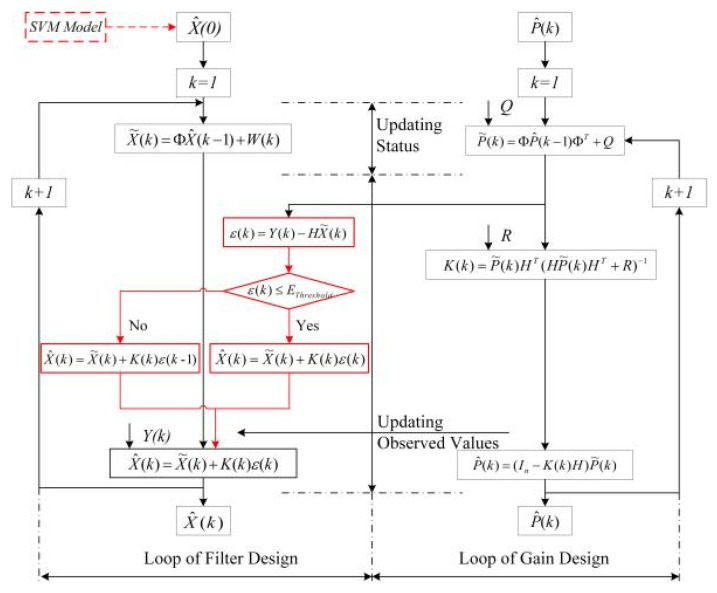
Flowchart for the improved Kalman filter.

To ensure that the target is kept track of, at least one node (node distributed in the radius $R_{S}$ activated by a communication protocol) in the predicted position $l_{k+1}^{\prime }$ at time $t_{k}$ can detect the target in position $l_{k+1}^{}$ at time $t_{k+1}$ [47]. That is to say, at least one node is deployed in the shaded area in Figure 3a. The communication and node wake-up protocols are not concerns in this study, but without a loss of generality, we can set $R_{X}=R_{S}$. Therefore, in the limit case, Figure 3a can be transformed to Figure 3b. As shown in Figure 3b, $E_{Threshold}\leq l_{k+1}^{}l_{k+1}^{\prime }$ in the case for which it is guaranteed that the target is not lost. Accordingly, we set $E_{Threshold}=l_{k+1}^{}l_{k+1}^{\prime }$ in this study. Since parameters $R_{S}$, $f_{k}$, and $\Delta t_{k}$ are predetermined and can be acquired, sin[θk2] can be calculated based on the knowledge of geometry, and only the speed change needs to be dynamically calculated in the online calculation of $E_{Threshold}$. This does not require an extensive number of calculations, and the computational complexity is low. The flowchart of our improved KF is shown in Figure 4.

## 4. Performance Evaluation and Analysis

### 4.1. Simulation Design

#### 4.1.1. Simulation Environment

In this section, we evaluate the performance of our method based on simulated data. Simulation experiments were carried out with MATLAB2017a installed on a PC with Intel(R) Core(TM) i5-8500 CPU@3.00 GHz and 16.00 GB RAM. We considered an area of 200 m×200 m and N nodes deployed within this area. To make our simulation experiments more viable and close to real-world scenarios, simulations were conducted under different environments, i.e., various node distributions, various anchor nodes, and various RSSI measurement noise. The RSSI values were obtained using the well-known Okumura–Hata model [20], given by

(23) PL(d)=Pr(d0)−10nlog(dd0)+Xσ,

where

- PL(d) is the signal path loss at distance d from the transmitter;
- $P_{r}(d_{0})$ is the RSSI measured at the receiver node located at the reference distance $d_{0}$ (generally $d_{0}=1m$);
- $X_{\sigma }$ is a normal random variable with a standard deviation of σ;
- n is the path loss exponent often set to a value of 4 [20].

Considering the error of the RSSI measurement value caused by NLOS and propagation loss in real applications, the RSSI measurement error between anchor nodes and the target was randomly generated with the $X_{\sigma }$. Equation (23) was also employed in the generation of off-line training data. Moreover, we set the target motion models with the constant velocity (CV) model and the constant acceleration (CA) model. Thus, X(k+1) in Equation (7) can be expressed as

(24) $$\{\begin{array}{c} X(k+1)=\Phi X(k)+\Gamma W(k)\begin{array}{cc} & CA,\;X(k)={\left[\overset{}{X}(k),\overset{•}{X}(k)\right]}^{T} \end{array}\\ X(k+1)=\Phi X(k)+\Gamma W(k)\begin{array}{cc} & CV,\;X(k)={\left[\overset{}{X}(k),\overset{•}{X}(k),\overset{••}{X}(k)\right]}^{T} \end{array} \end{array},$$

where

- $\overset{}{X}(k),\overset{•}{X}(k),\overset{••}{X}(k)$ are the displacement, velocity, and acceleration of the target, respectively, and
- Γ is the system control matrix.

#### 4.1.2. Performance Metrics

We evaluated the tracking system in terms of the accuracy, precision, stability, and computational time. The tracking precision measures the difference between the estimated (or predicted) and the actual target’s position, and the root-mean-square-error (RMSE) was employed as a metric. The RMSE of the 2D target position can be calculated as

(25) $$RMSE=\sqrt{\frac{1}{n}\sum_{i=1}^{n}\left({\left(\bar{x}(i)-x_{0}(i)\right)}^{2}+{\left(\bar{y}(i)-y_{0}(i)\right)}^{2}\right)},$$

where

- $\left(x_{0}(i),y_{0}(i)\right)$ and $\left(\bar{x}(i),\;\bar{y}(i)\right)$ are the actual and estimated positions calculated by the TT algorithm at time i, respectively, and
- n is the number of samples.

To evaluate the tracking algorithm more comprehensively, we employed another metric defined as the success probability of position calculation with respect to a predefined accuracy. We applied the Cumulative Distribution Function (CDF) of the localization error to evaluate our system. The localization error can be calculated by

(26) $$L{\mathrm{ocalization}}_{}\;error_{i}=\sqrt{{\left(\bar{x}(i)-x_{0}(i)\right)}^{2}+{\left(\bar{y}(i)-y_{0}(i)\right)}^{2}}.$$

To compare the stability of the algorithm, the variance value of localization error was also selected as one of the metrics, which can be obtained by

(27) $$V\mathrm{aricance}=\frac{1}{n}\sum {\left(L{\mathrm{ocalization}}_{}\;error_{i}-L{\mathrm{ocalization}}_{}\;error_{average}\right)}^{2}.$$

#### 4.1.3. Parameter Setup

In the offline data preprocessing stage, the settings of parameters Eps and MinPts determine the effect of the DBSCAN algorithm on the elimination of RSSI outliers. In this study, we determined that the values Eps=3 and MinPts=3 were ideal based on a large number of simulations and verifications.

In the training of the SVM model, the optimal combination of the penalty coefficient C and kernel function parameter γ was determined by a nested cross-validation method [48,49]. The optimal (C,γ) in this study was set to C=2700,γ=0.005 after training. In addition, the default parameter settings of the KF at time T are listed in Table 1.

### 4.2. Simulation Results and Analysis

We compared the simulation results of our method with those obtained from five relevant algorithms—the traditional KF; our improved KF; the PF algorithm in [49]; and two relevant LBE methods, consisting of the RR + KF presented in [20] and the GRNN + KF presented in [36]. Brief descriptions of the algorithms are summarized in Table 2.

#### 4.2.1. Evaluation over Different Trails

**Figure 5:**
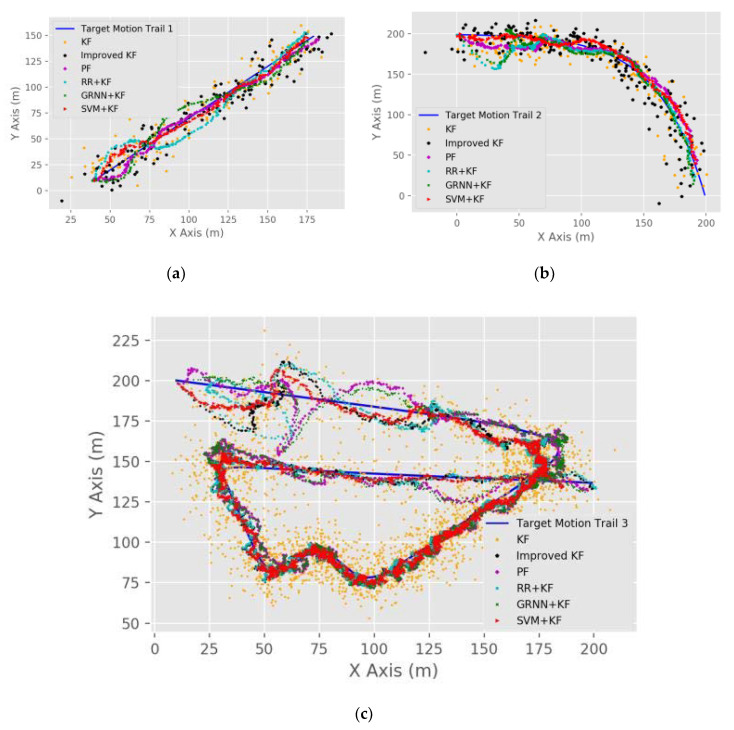
Results of different target motion trails: (**a**) Simulation results of trail 1; (**b**) simulation results of trail 2; (**c**) simulation results of trail 3.

**Figure 6:**
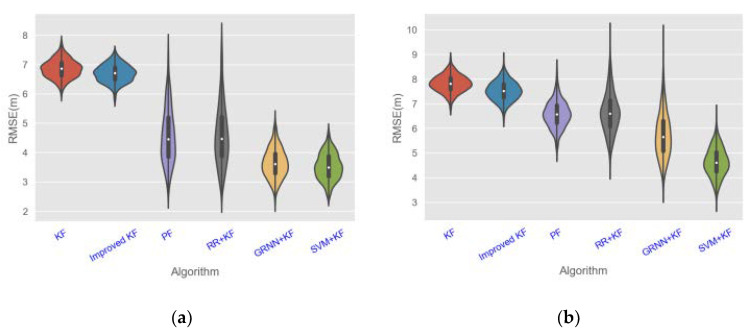

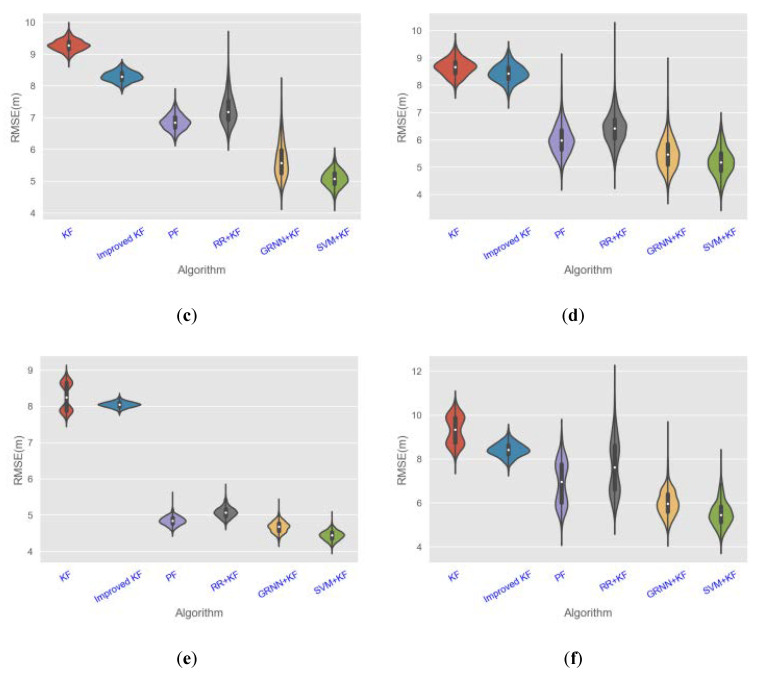
Violin diagram of simulation results of different algorithms with 100 rounds. **a**, **c**, and **e** are the simulation results of motion trail 1, 2, and 3 in the constant velocity (CV) motion model, respectively, and **b**, **d**, and **f** are the simulation results of motion trail 1, 2, and 3 in the constant acceleration (CA) motion model, respectively.

We considered three different target motion trails. The first two trails were used to simulate the target’s simple linear and curved motions, and the third one had multiple bends and irregular changes and was thus more complicated than the other two. This enabled us to test the performance of each method in different scenarios. The parameter settings used in the simulations are summarized in Table 3. Some of the simulation results are shown in Figure 5. To facilitate a numerical comparison of the performances of different algorithms, simulation results with 100 runs are illustrated in Figure 6 based on a violin diagram.

As can be seen in Figure 6, in general, the RMSE values in trails 1 and 2 of each algorithm are smaller than that in trail 3, and the RMSE values of each algorithm in the CA motion model are smaller than those in the CV motion model. Figure 6 illustrates that SVM + KF has the highest tracking accuracy, as the mean value of RMSE of SVM + KF (2.1–8.3 m) is 3.126%, 5.379%, 9.024%, 10.054%, and 12.287% lower than GRNN + KF (1.9–10.3 m), RR + KF (2.1–11.2 m), PF (3.9–12.3 m), Improved KF (5.3–9.7 m), and KF (5.4–11.3 m), respectively.

**Figure 7:**
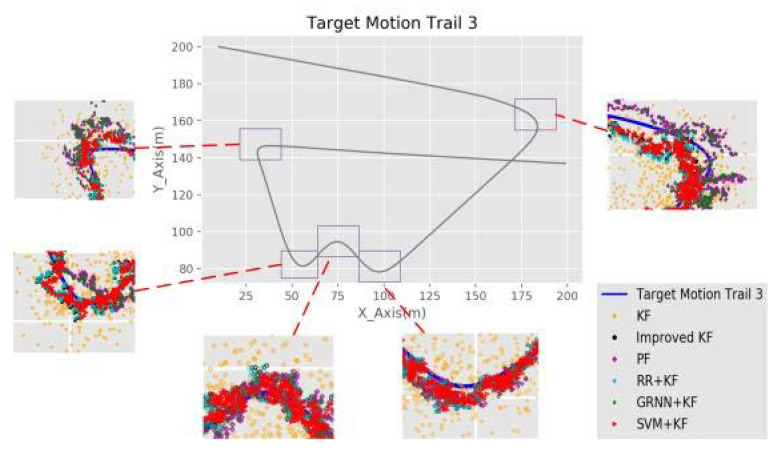
Simulation results in the turning region of trail 3.

The original motion models (CV and CA) are not designed to describe the motion of the target when the target turns. This constitutes a major challenge for the target positioning accuracy. In this case, the tracking performances of all the algorithms are usually much worse. Figure 7 shows the TT results for each algorithm in the turning region of trail 3. We chose the RMSE value and the value of the variance as metrics to evaluate the accuracy and stability. The simulation results for each algorithm in the turning region of trail 3 with 100 rounds are listed in Table 4. 

Table 4 illustrates that when the target turns, the RMSE and variance values of SVM + KF are the lowest. Nevertheless, more suitable models can be used to describe the turns of the target to achieve better results. Considering that the SVM + KF method combines the nonlinear expression ability of SVM and the stability improvement of the new KF, we can consider that the tracking accuracy and stability of SVM + KF are optimal for a given trail and motion model. 

Moreover, the RMSE of the SVM + KF is similar to GRNN + KF, but the stability of SVM + KF is significantly improved (in the range of 10–20%) compared with GRNN + KF. This is primarily because the improved KF (in Section 3.2.2) of our algorithm uses the innovation threshold to limit the interference attributed to the RSSI outliers, which leads to a substantial improvement in the stability of the TT result.

**Figure 8:**
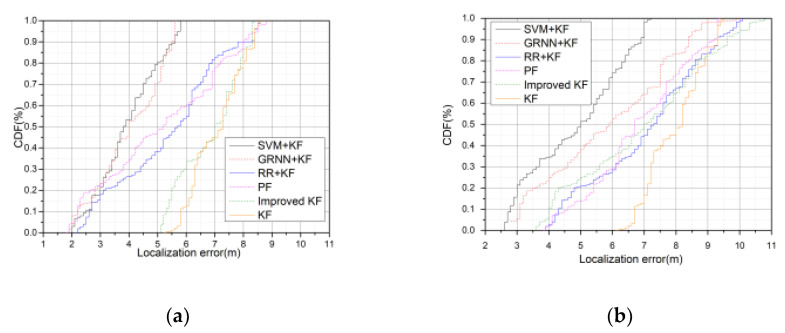

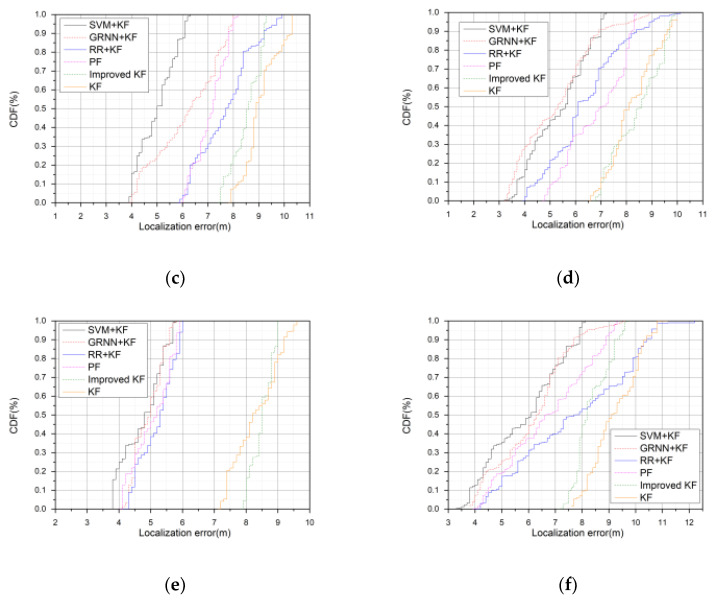
Comparison of Cumulative Distribution Function (CDF) localization error between different algorithms with 100 rounds. **a**, **c**, and **e** are the simulation results of motion trail 1, 2, and 3 in the CV motion model, respectively, and **b**, **d**, and **f** are the simulation results of motion trail 1, 2, and 3 in the CA motion model, respectively.

Figure 8 illustrates the CDF of the localization error of different algorithms. In general, the CDF values for trails 1 and 2 of each algorithm are bigger those for trail 3, and the CDF values of each algorithm in the CA motion model are bigger than those of the CV motion model. The figure illustrates that 95% of the localization errors of SVM + KF, GRNN + KF, RR + KF, PF, Improved KF, and KF are below 5.2–6.1, 5.4–6.9, 5.3–9.3, 5.9–10.1, 7.8–10.2, and 8.4–10.7 m, respectively. 

#### 4.2.2. Impact of the Number of Anchor Nodes

**Figure 9:**
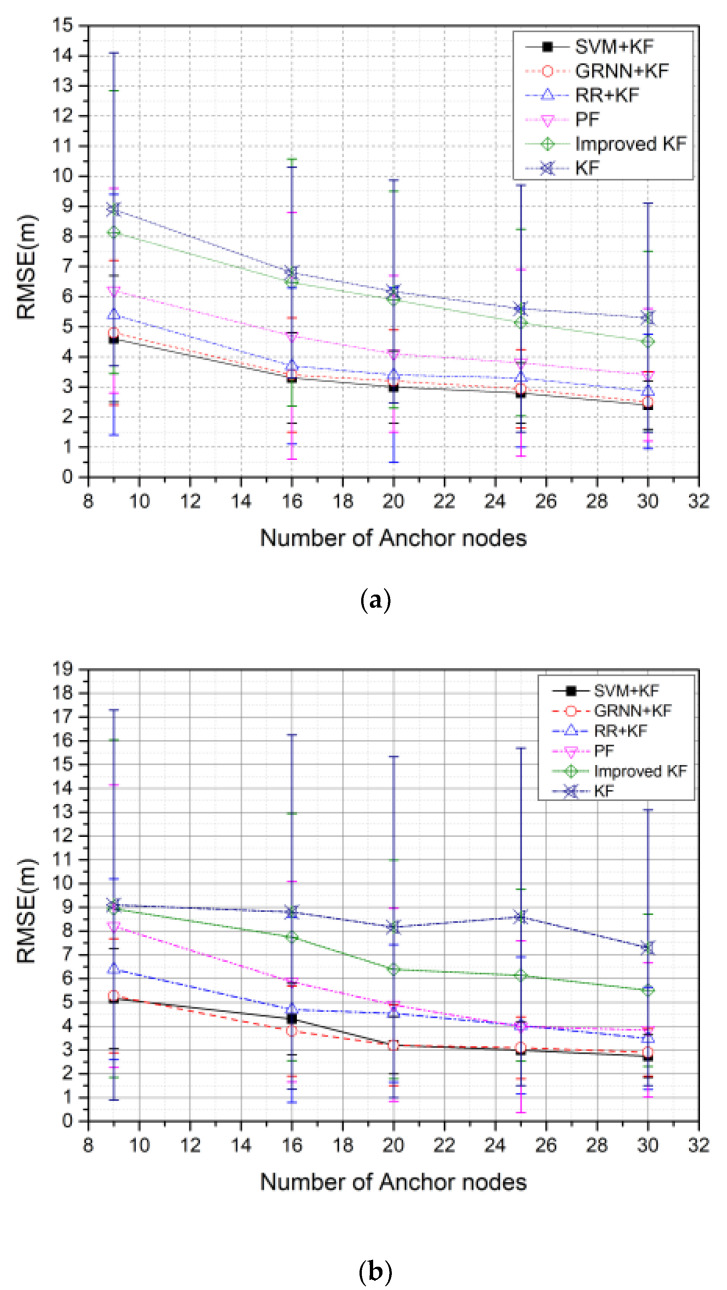
RMSE versus number of anchor nodes with 100 rounds: (**a**) Anchor nodes with a uniform distribution; (**b**) anchor nodes with a random distribution.

We then analyzed the impact of the number of anchor nodes, using trail 3 in the CA motion model, which is a more representative simulation environment for practical scenarios. We first uniformly deployed a simulation with a number of anchor nodes in the surveillance area. We then considered a random distribution, instead of a uniform distribution, to examine the impact of such a choice on our method. The parameter settings in the simulations are summarized in Table 5. Figure 9 illustrates the effect of the number of anchor nodes on the RMSE of the algorithms under uniform and random distributions, deployed with 100 rounds.

As shown in Figure 9, the RMSE values of the six algorithms decrease as the number of nodes increases. Figure 9a illustrates that SVM + KF has the highest localization accuracy, as the mean RMSE values of SVM + KF are 3.254%, 4.819%, 7.297%, 10.054%, and 11.395% lower than GRNN + KF, RR + KF, PF, Improved KF, and KF, respectively. Moreover, Figure 9b illustrates that SVM + KF has the highest localization accuracy, as the mean RMSE values of SVM + KF are 3.821%, 5.7889%, 8.406%, 11.116%, and 12.701% lower than GRNN + KF, RR + KF, PF, Improved KF, and KF, respectively.

Comparing Figure 9 and b, we can see that the algorithms perform better when the anchor nodes are uniformly distributed, compared to when they are randomly distributed. SVM + KF is slightly better than GRNN + KF in terms of the accuracy of the tracking results, but the stability of the former is considerably higher than that of the latter. This benefit of stability offered by SVM + KF primarily stems from the fact that our improved KF (Section 3.2.2) uses the innovation threshold to enhance the stability of the tracking results.

#### 4.2.3. Impact of RSSI Measurement Noise

**Figure 10:**
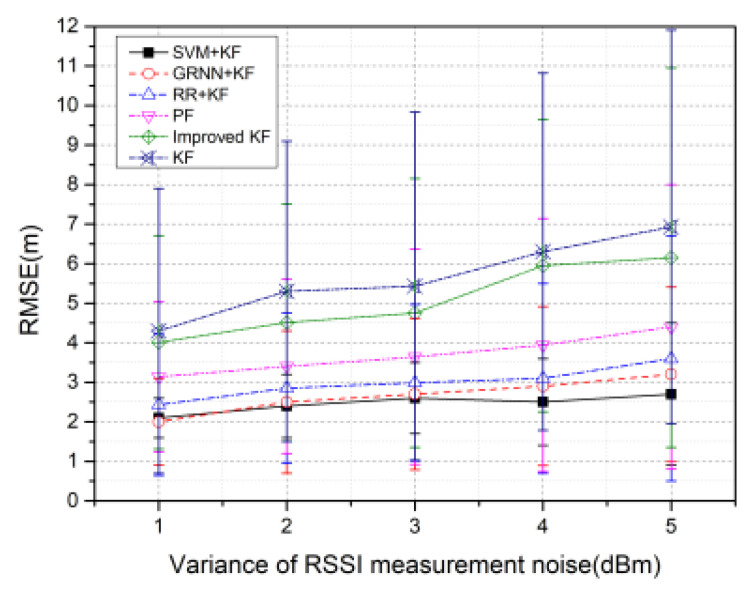
RMSE versus variance of received signal strength indication (RSSI) measurement noise with 100 rounds.

Figure 10 illustrates the changes in the RMSE values of the algorithms over different RSSI measurement noise values with 100 rounds. The parameter settings in the simulation are summarized in Table 6. 

As shown in Figure 10, the RMSE values of the six algorithms gradually increase as the variance of RSSI measurement noise increases. In most cases, SVM + KF has the highest tracking accuracy, as the mean value of RMSE of SVM + KF is 2.504%, 3.667%, 6.717%, 9.071%, and 11.295% lower than GRNN + KF, RR + KF, PF, Improved KF, and KF, respectively.

#### 4.2.4. Computational Time

To examine the runtimes of the different algorithms, the time taken to compute a single mobile target’s per step tracking was calculated for each of the algorithms. The results are summarized in Table 7. Since the prelocalization computed by pattern matching algorithms consumes a certain amount of time, the LBE algorithms (GRNN + KF, RR + KF, and SVM + KF) run slower than the KF, Improved KF, and PF algorithms. Nevertheless, although SVM + KF is relatively more complex (primarily due to the dynamic setting of the innovation threshold) than GRNN + KF and RR + KF, their running times only marginally differ.

#### 4.2.5. Analysis

Generally, the tracking performances of the algorithms are better in the simpler trails 1 and 2, than in the more complicated trail 3, and the performances of the algorithms are better in the CA motion model than in the CV motion model. SVM + KF exhibited the highest tracking accuracy, and its stability is better than those of the other algorithms. Moreover, by comparing it with the KF and our improved KF algorithms, we can see that each step of the SVM + KF algorithm contributes to the improvement of the accuracy and stability of the tracking results, especially in the non-Gaussian noise environment. 

Although RR + KF, GRNN + KF, and SVM + KF are all LBE methods and trained by the same dataset, the SVM + KF method performs better in terms of the accuracy and stability compared to the other two. This can be attributed to the superior nonlinear processing ability of SVM and the improved KF method, which significantly enhances the stability and ensures accuracy. While SVM + KF is slightly more complex, the running time it requires is comparable to that of the other two LBE methods.

Furthermore, the performance of SVM + KF is substantially higher than that of the PF. This is due to the phenomenon of particle fading in PF, which results in a lower stability than our method. In contrast, SVM + KF draws from the combined ability of the SVM and the improved KF to precisely predict the position of the target, and is therefore able to alleviate this problem with a stable performance.

### 4.3. Experiments 

In order to verify and validate the performance of the SVM + KF TT algorithm in real-world scenarios, we deployed the algorithm in practical experiments using a WSN experimental platform. We also compared the performance of SVM + KF in the experiment with the same LBE, RR + KF [20], and GRNN + KF [36] algorithms used in the simulations. 

#### 4.3.1. Experimental Design

**Figure 11:**
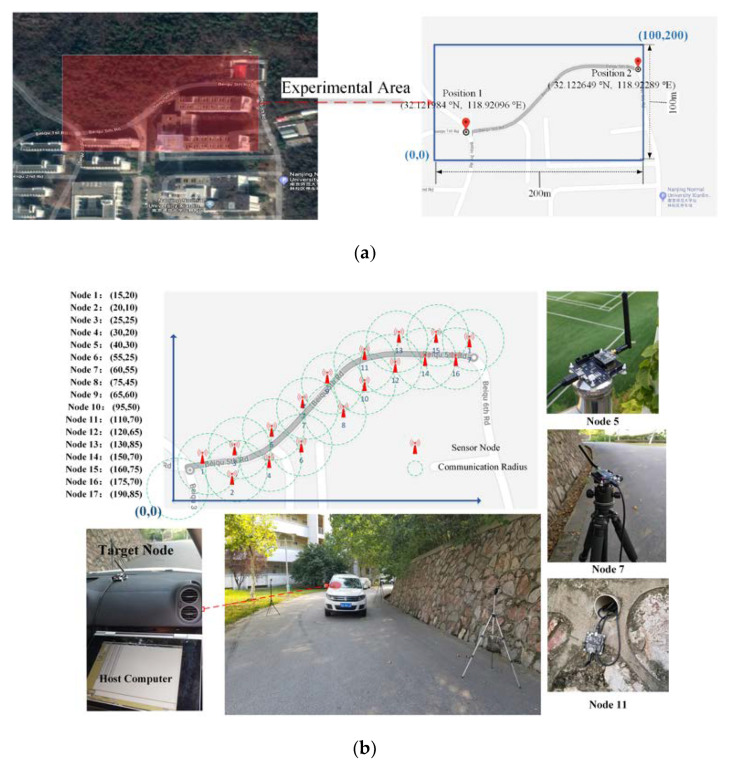
Photograph of experiments: (**a**) Experimental area in GoogleMap; (**b**) deployment of equipment.

We chose Beiqu’s 5th Road in the Qixia Campus of Nanjing Normal University as the experimental area, which has a size of 100 m×200 m, as shown in Figure 11a. For this area, the flat terrain can be approximated as 2D planes, thus making it suitable for the needs of the experimental scenario. 

In total, 17 sensor nodes were used to form a WSN supervised area, as shown in Figure 11b. We primarily used the optimal, complete coverage method proposed in [50] as our strategy for the deployment of sensor nodes. Each sensor node had its own position coordinates. Furthermore, all of the sensor nodes were placed at a height of 1 m. The sensor nodes were designed with the CC2530 F256 digital signal processing (DSP) starter kit of Texas Instruments. The main parameters of the chip were as follows: the working frequency band was 2.4 GHz, the receiving sensitivity was less than -100 dBm, the Zigbee protocol was carried for data packet transmission and communication, and the transmission distance threshold was 30 m. Since the wake-up mechanism of nodes was not the focus of this study, all of the nodes were running during the experiment. 

#### 4.3.2. Experimental Process 

We used 800 RSSI signals collected in different scenarios for offline SVM model training. The entire online tracking experimental process is described as follows:

- Firstly, the sensor nodes periodically broadcasted information after deployment;
- Secondly, the target node was placed in a car, which served as the information collection center; it also carefully received measurements from the sensor nodes and transmitted them to the host computer (the above operations were also used for the collection of training data, and the collected data was then preprocessed with DBSCAN, and its data cleaning performance was evaluated by checking whether the “noise points” calculated by DBSCAN were accurate);
- Finally, the host computer was used to implement different TT algorithms, and the results were analyzed and evaluated based on comparisons with the target’s ground truth trajectories.

In order to accurately measure the target’s ground truth trajectories, we utilized global positioning system (GPS) devices for positioning, and used the road’s centerline as a reference for the target’s (car) maneuvers. To synchronize the sampling time of the WSNs, the actual position at each sampling time was calculated by an interpolation method based on the position and time.

To numerically compare the performance of the RR + KF, GRNN + KF, and SVM + KF algorithms, we calculated the average RMSE value (hereinafter referred to as the RMSE), and the variance of the three LBE algorithms based on 50 trajectories. During each tracking experiment, instead of using the motion modes (CA and CV), the target (car) ran with a random velocity in the range of 30 to 80 km/h. 

**Figure 12:**
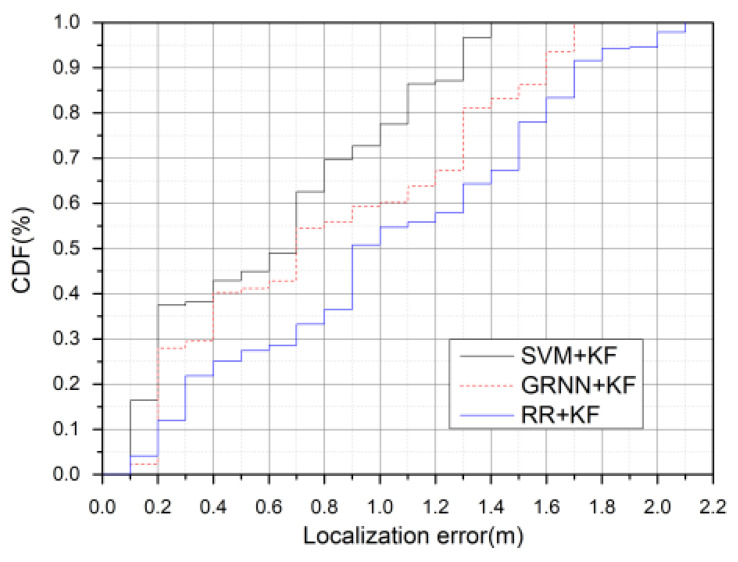
Comparison of CDF localization error between different learning-by-example (LBE) algorithms.

**Figure 13:**
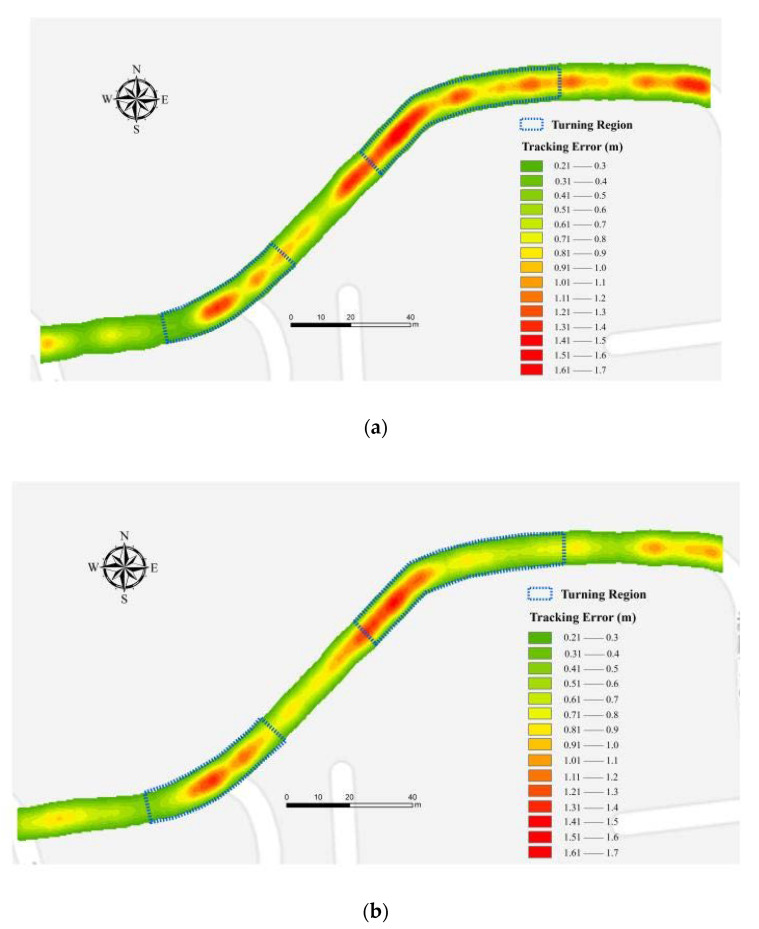

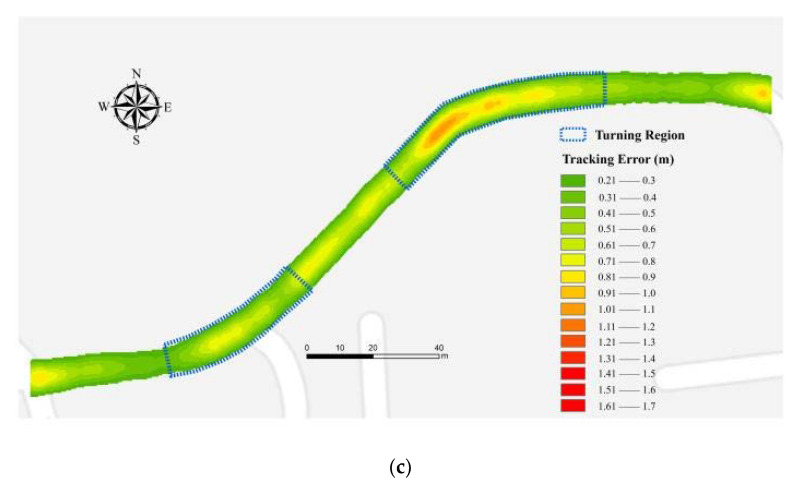
Error distribution of each algorithm: (**a**) ridge regression (RR) + Kalman filter (KF); (**b**) generalized regression neural network (GRNN) + KF; (**c**) SVM + KF.

To facilitate a comprehensive analysis, we listed the tracking results in the turning area of each algorithm according to the conclusions inferred based on the simulation tests in Section 4.2.1. The comparison of CDF localization error for different LBE algorithms is shown in Figure 12, the error distribution of each algorithm is shown in Figure 13, and the statistics obtained from the experimental results of each algorithm are shown in Table 8.

#### 4.3.3. Results and Analysis

Figure 12 illustrates that 95% of the localization errors of SVM + KF, GRNN + KF, and RR + KF, are below 1.3, 1.6, and 1.8 m, respectively. Combining the results from Figure 13 and Table 8, we can see that the decrease of the accuracy in the turning area varies between the LBE algorithms. In terms of the tracking accuracy, the results in Table 8 illustrate that the performance of SVM + KF (0.87 m) increases by more than 38% and 30% compared to RR + KF (1.41 m) and GRNN + KF (1.24 m), respectively, in the entire trajectory. Table 8 also reveals that the RMSE for the turning region in our algorithm is less than 1 m, and improves by 37% over RR + KF (1.55 m) and 26% over GRNN + KF (1.33 m). The variance of SVM + KF is the lowest in both the entire trajectory and the turning region, with an improvement of 47% compared to RR + KF and 34% compared to GRNN + KF in the turning region, and an improvement of 34% over RR + KF and 20% over GRNN + KF over the entire trajectory.

### 4.4. Discussion

The simulation results demonstrate that our algorithm provides a superior performance in comparison with other algorithms in terms of the accuracy and stability in various scenarios. The practical experiments, in which the RMSE of the tracking results reduced by 0.87 m using the SVM + KF algorithm, further confirm these conclusions. Nevertheless, tracking in turning regions constitutes a major challenge in target tracking. While a better motion model might significantly improve the tracking accuracy, our algorithm provided the best results with the standard motion models used in our simulations and experiments. 

In comparison with the two LBE methods studied here, SVM + KF achieved an optimal tracking performance in most cases. SVM + KF was able to particularly improve the stability of the tracking results to a substantial extent. While this improvement is primarily attributable to the dynamic setting of the innovation update threshold in KF (Section 3.2.2), it results in a marginally higher running time. While, in this study, we only considered combining SVM with KF, our proposed TT approach can be extended to incorporate other filtering methods, such as EKF, UKF, and PF, potentially leading to further gains in the tracking accuracy.

## 5. Conclusions

In this study, we proposed a new TT method, known as the SVM + KF method, for WSN applications. The SVM + KF method avoids the establishment of a path-loss model. Instead, it uses the SVM method to establish the relationship between the position of the target and the RSSI value received by the nodes in a WSN to estimate the target’s position. This estimate is then corrected by an improved KF method based on innovation modification to achieve a higher accuracy. Simulation and experimental results demonstrated that the proposed method effectively improved the tracking accuracy and stability in comparison with other relevant algorithms. The method allowed accurate tracking and was proved to be robust with nonlinear non-Gaussian noisy data, and therefore provides a useful contribution to applications of KF in RSSI-based TT.

## Figures and Tables

**Table 1:** Default parameter settings of the Kalman filter at time T.

	CV	CA
Φ in Formula (7)	$\left[\begin{array}{ll} 1 & T\\ 0 & 1 \end{array}\right]$	$\left[\begin{array}{lll} 1 & T & 0.5T^{2}\\ 0 & 1 & T\\ 0 & 0 & 1 \end{array}\right]$
Γ in Formula (24)	$\left[\begin{array}{c} 0.5T^{2}\\ T \end{array}\right]$	$\left[\begin{array}{c} 0.5T^{2}\\ T\\ 1 \end{array}\right]$
H in Formula (8)	$\left[\begin{array}{cc} 0 & 1 \end{array}\right]$	$\left[\begin{array}{ccc} 0 & 0 & 1 \end{array}\right]$
Sampling interval	1s	1s

**Table 2:** Details relevant to the comparison of used algorithms.

Algorithms	Description
KF	Traditional KF algorithm described in Section 3.1.2
Improved KF	Improved KF based on innovation modification in Section 3.2.2
PF	PF-based algorithm presented in [49]
RR + KF	Algorithm presented in [20]
GRNN + KF	Algorithm presented in [36]
SVM + KF	Our proposed algorithm

**Table 3:** Parameter settings.

Parameters	Value
Anchor node (N)	25
Anchor nodes distribution	Uniform
Variance of RSSI measurement noise (σ in Formula (23), where mean = 0)	2
Motion model	[CV, CA]
Motion trail	1,2,3

**Table 4:** Simulation results of different algorithms in the turning region of trail 3 with 100 rounds.

		KF	Improved KF	PF	RR + KF	GRNN + KF	SVM + KF
CA	RMSE	6.699	6.856	8.757	4.637	3.550	3.541
	Variance	1.133	1.082	0.458	0.313	0.322	0.289
CV	RMSE	8.749	8.428	7.704	6.619	5.876	5.852
	Variance	2.020	0.521	0.584	0.593	0.387	0.301

**Table 5:** Parameter settings.

Parameters	Value
Anchor node (N)	9,16,20,25,30
Anchor nodes distribution	[Uniform, Random]
Variance of RSSI measurement noise (σ in Formula (23), where mean = 0)	2
Motion model	CA
Motion trail	3

**Table 6:** Parameter settings.

Parameters	Value
Anchor node (N)	25
Anchor nodes distribution	Uniform
Variance of RSSI measurement noise (σ in Formula (23), where mean = 0)	1,2,3,4,5
Motion model	CA
Motion trail	3

**Table 7:** Running times of the algorithms.

Algorithms	Running Times (s)
KF	0.000517
Improved KF	0.000829
PF	0.457428
RR + KF	0.905721
GRNN + KF	0.919547
SVM + KF	0.922715

**Table 8:** Statistics obtained from experimental results.

		RR + KF	GRNN + KF	SVM + KF
Entire trajectory	RMSE	1.41	1.24	0.87
	Variance	0.86	0.71	0.57
Turning region	RMSE	1.55	1.33	0.98
	Variance	1.23	0.97	0.64
